# Prevalence of adult eczema, hay fever, and asthma, and associated risk factors: a population-based study in the northern Grassland of China

**DOI:** 10.1186/s13223-021-00532-7

**Published:** 2021-03-09

**Authors:** Xiaoyan Wang, Yan Zhuang, Yanlei Chen, Hongtian Wang, Xueyan Wang

**Affiliations:** 1 Department of Allergy, Beijing Shijitan Hospital, Capital Medical University, No. 10, Tieyi Road, Haidian District, Beijing, 100038 China; 2 Allergy Center, Beijing Shijitan Hospital, Capital Medical University, Beijing, 100038 China

**Keywords:** Eczema, Atopic dermatitis, Socioeconomic status, Risk factors, Allergen sensitization

## Abstract

**Background:**

There has been research about the prevalence and risk factors of eczema, hay fever, and asthma in children, but little is known about these conditions in adults in China.

**Objectives:**

To explore the prevalence of adult eczema/atopic dermatitis (AD) and its risk factors in northern China.

**Methods:**

A cluster sampling randomized population-based survey was conducted using a face-to-face questionnaire combined with skin prick tests of ten common aeroallergens including nine pollen allergens and *Dermatophagoides pteronyssinu* (Dp) allergen. The questionnaire was designed by specialists and included questions on the prevalence of eczema, hay fever, and asthma, socioeconomic risk factors, family history of atopy and environmental exposures. The prevalence of eczema with asthma and/or hay fever (EAH) was applied as a proxy of AD in this study.

**Results:**

Overall, 2096 subjects were enrolled and completed the study. The prevalence of eczema was 15.7% (95% CI 14.3–17.4), while the prevalence of hay fever and asthma were 20.6% (95% CI 18.9–22.4) and 6.5% (95% CI 5.5–7.6), respectively. In particular, the prevalence of EAH was 5.1% (95% CI 4.4–7.0). The prevalence of eczema and EAH was significantly associated with younger age, atopy family history, high education level, urbanization, and antibiotic overuse (*P* < 0.05, logistic regression). The sensitization rate was higher in EAH compared with eczema (48.2% vs 41.0%, *P* = 0.018), with weed pollen sensitization being the most common. Subjects with two or more concomitant allergic diseases had increased risk of eczema and EAH (*P* < 0.001). Allergen sensitization increased the risk of eczema and EAH (*P* < 0.001, both).

**Conclusions:**

Adult eczema and EAH are prevalent in northern China under high pollen exposure. Socioeconomic and environmental factors affected the prevalence of adult AD in China. Dp had a particular impact on the prevalence of eczema/AD in the grassland region.

## Introduction

Atopic dermatitis (AD) is a chronic, inflammatory cutaneous disorder that typically begins in early childhood, with a prevalence of 7–10% [1, 2]. AD is associated with several comorbidities and clinical manifestations such as skin lesions that profoundly alter quality of life in adults [3]. AD has been associated with anxiety, depression and suicidal ideation and imparts a large burden in terms of both reduced work productivity and increased global health care expense [4, 5].

Traditionally, AD is considered a disease of childhood that remits in adolescence and adulthood [2, 6]. Although 60–75% eventually outgrow AD with age, some continue to experience AD symptoms in adulthood [6–8]. Furthermore, one in four adults with AD report adult-onset disease [6, 8]. Globally, most studies focus on the prevalence of AD in children [4, 9–15]. Surprisingly, few studies looked at the epidemiology of AD in adults. Studies in the US, Europe, Japan, Italy, and Sweden demonstrated varying disease prevalence, showing a lower prevalence of adult AD compared with childhood AD [16–18] However, few such studies have been conducted in China. Adult AD usually deviates from the classic pattern of childhood AD, and is associated with distinct risk factors, clinical feature, associated risk factors, genetics, and comorbidities. Epidemiologic studies are needed in adult populations to determine the features of adult AD.

The diagnosis of adult AD is challenging [19, 20]. The clinical criteria of AD, such as the Hanifin and Rajka criteria, are impractical for large-scale surveys. Other criteria, such as the UK Working Party criteria, Japanese Dermatological Association criteria, and Eczema Prevalence Impact Working Group diagnostic criteria, were also applied in different surveys [6, 21]. Due to the inability to accurately diagnose AD across a large population in a cost-effective way, the diagnosis of adult AD requires standard criteria. In recent years, the comorbidity of atopic diseases (asthma and/or hay fever) with eczema have been defined as EAH and was employed in several epidemiological studies to represent AD [17, 22]. Each definition captures a different subset of the eczema/AD disease spectrum, and therefore both definitions were included in our population-based study.

The prevalence of childhood AD is reported to be 2.5–12.94% in China [23–25]. To our knowledge, there are no recent studies on adult AD in China. The aim of this study was to explore the prevalence and determinants of adult eczema/AD in the northern grassland region of China using data obtained from a face-to-face questionnaire. The clinical data we obtained from this survey provide information such as risk factors and allergic sensitization status of adult AD in a region under high pollen exposure according to EAACI’s definition [26] and our prior study [27]. This information provides advice regarding lifestyle and prevention of adult AD in this region.

## Methods

### Study design

**Fig. 1:**
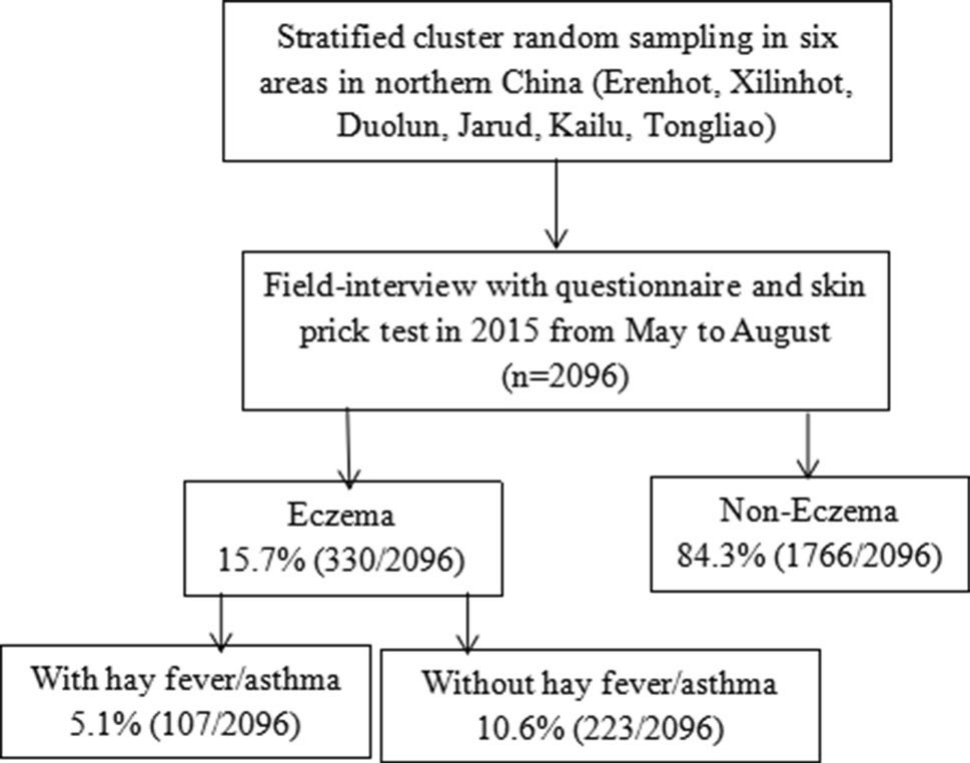
The flow chart of the survey

**Fig. 2:**
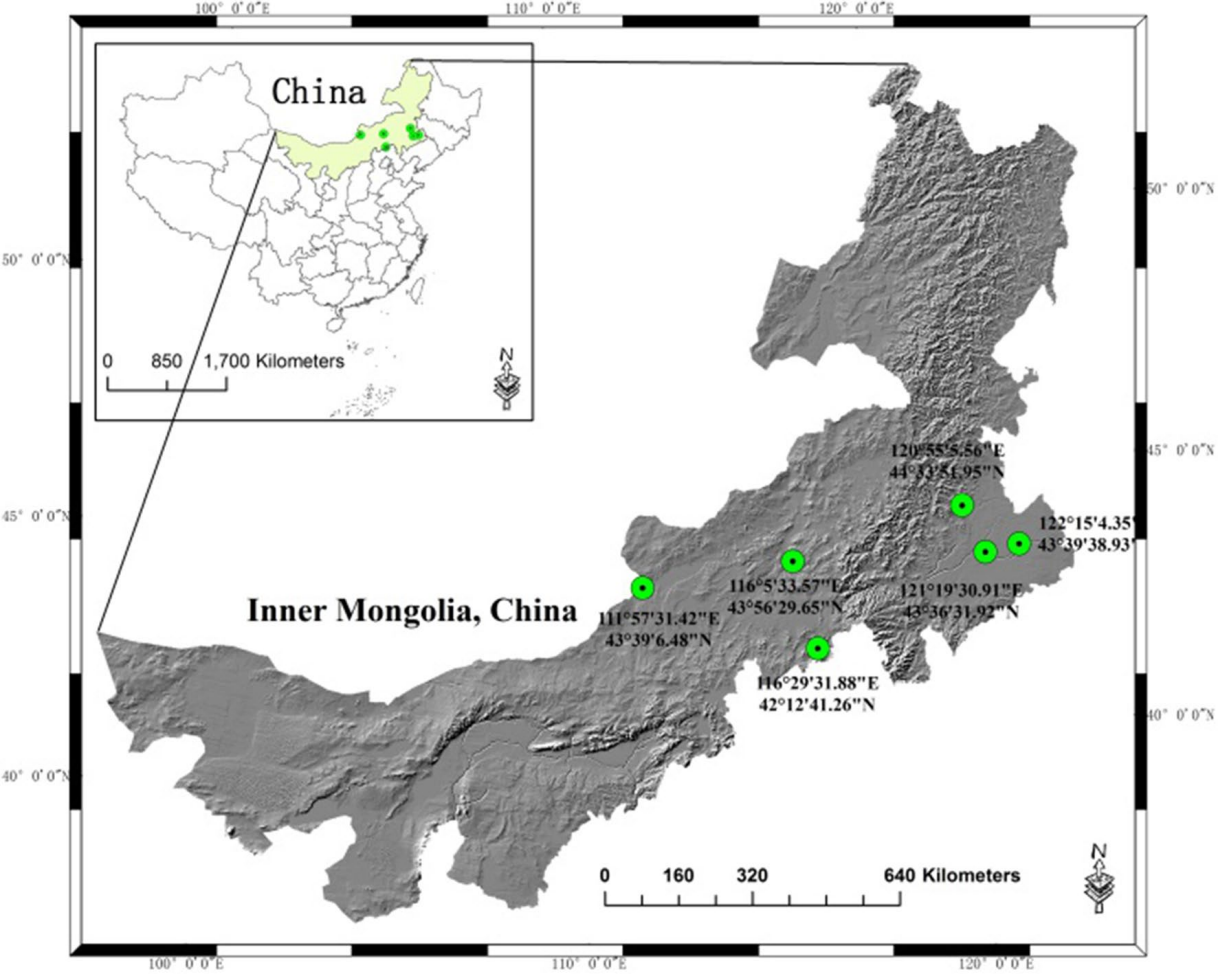
Geographic locations of the study sites, including the Erenhot, Xilinhot, Duolun, Tongliao, Jarud, and Kailu areas of northern China

A multistage, clustered, stratified survey was carried out in the northern grassland of China from May to August of 2015 (Fig. 1). The timeline was alos the pollen season locally. The study was designed by a group of epidemiologists. The geographic location of survey activity is found in Fig. 2. The Xilingol and Horqin grasslands, which are located in the eastern and middle part of the Inner Mongolia Autonomous Region of China, were included, and six study regions (Erenhot, Xilinhot, Duolun, Jarud, Kailu, and Tongliao) were selected (Fig. 2). The survey was conducted by the same team sequentially to maintain the uniformity. The interviewers consisted of specialists and nurses who were trained before the investigation. The participants were chosen by random clustering method and informed at least two weeks earlier either by a letter or by a telephone call by local coordinators or healthcare officers. All subjects from the selected clusters were invited to finish this study.

### Questionnaire data

All subjects received a face-to-face interview conducted by trained specialists using a standard questionnaire. The questionnaire included demographic information such as age, gender, ethnic group, place of residence, education level, socioeconomic status, family income, and body mass index (BMI). Data regarding allergic disorders (hay fever, conjunctivitis, asthma, food allergy, and drug allergy) and concomitant chronic diseases, family history of atopy, and environmental risk factors were also collected. The questionnaire was double-checked and re-answered if the quality was unsatisfactory. All participants signed informed consent. For those < 18 years, informed consent was signed by their parents or guardians. Approval to conduct this study was granted by the institutional review boards of Beijing Shijitan Hospital, the affiliated hospital of the Beijing Capital Medical University (2015 No. 3).

### Disease definitions

Eczema was defined as a positive response to the following questions: “During the past year, have you had dermatitis, eczema, or any red, inflamed skin rashes?” or “Have you been diagnosed with eczema or dermatitis by a dermatologist during past year?” or typical eczema rashes were found on the study participants.

Hay fever was defined as a positive response to the following questions: “During the past year, have you been diagnosed with hay fever by a doctor or health professional?” or “Did you have at least two of the four common symptoms (itchy nose, sneezing, runny, and blocked nose) for at least one hour on pollen days during the past year?”.

Asthma was defined as a positive response to the following question: “Have you been diagnosed with asthma by a physician during the past year?”.

Subjects who were reported to have eczema combined with asthma and/or hay fever were defined as having EAH as published [15, 16, 21]. EAH has been used to represent AD in previous studies.

### Skin prick test

All participants received skin prick tests (SPTs) of the ten common aeroallergens in the northern grassland region, which were determined by a combination of pollen monitoring and our preliminary study [27]. The ten common aeroallergens were: *Artemisia *(*Ar*)*; Betula* (Be)*, Chenopodium *(*Ch*); *Humulus scandens *(*Hu*); *Salix *(*Sa*); *Zea mays *(*Ze*); *Juniperus *(*Ju*); *Ulmus pumila *(*UI*); *Populus *(*Po*), and *Dermatophagoides pteronyssinus* (*Dp*). Standardized allergen extracts (Macro-Union Pharmaceutical Lim, Beijing, China) were applied. Antihistamines and other related medications were restricted at least three days prior to SPT. SPT was performed by experienced nurses following a standard protocol [28]. The drop of aeroallergen was placed on the flexor side of the forearm, and then the central part of the allergen was pricked using a sterile skin prick needle. The puncture depth was restricted to the epidermis. The distance between each allergen was 2 cm. Histamine and saline were applied as positive and negative controls, respectively. The results were evaluated 15 min after the procedure. A wheal diameter ≥ 3 mm was regarded as positive, whereas < 3 mm was considered negative. In addition to analyze the severity of sensitization, a positive score ranged from class 1 to class 4 was defined based on the wheal size. Class 1: a wheal diameter between 3 and 5 mm; Class 2: a wheal diameter between 5 and 10 mm; Class 3: a wheal diameter between 1 and 2 cm; Class 4: a wheal diameter ≥ 2 cm and present with pseudopods.

### Covariates and confounders

The covariates in this study were recorded from the questionnaire. Socioeconomic factors: gender; age group; education status (primary education, high school education, or graduated from college or university); family income; and BMI, with BMI < 25 kg/m^2^ defined as lean or healthy, BMI between 25 and 29 kg/m^2^ defined as overweight, and BMI ≥ 30 kg/m^2^ defined as obese. Environmental risk factors: current smoking status (non-smoker, smoker, ex-smoker), pet keeping, heating mode (wood, coal, or central heating), overuse of antibiotics (> 3 times/year), and outdoor activity (≤ 1 h, 2–3 h, ≥ 4 h).

### Statistical analyses

Data were processed and analyzed with IBM SPSS Statistics for Windows Version 23.0 (IBM Corp, Armonk, NY, USA) by a professional biostatistician. Categorical data ware described as numbers and percentages. Continuous data are shown as mean ± standard deviation (SD). Differences between groups in subject characteristics were tested using a *t* test or Wilcoxon rank-sum test for continuous variables, and chi-square or Fisher exact test was used for categorical variables. The Bonferroni correction was performed to correct *P* values of multiple pairwise comparisons. Multivariate logistic regression analysis was performed to explore the risk factors related to adult eczema and estimate their odds ratios. All tests were two-sided with a significance level of 0.05.

## Results

### Prevalence of adult eczema and hay fever/asthma

**Fig. 3:**
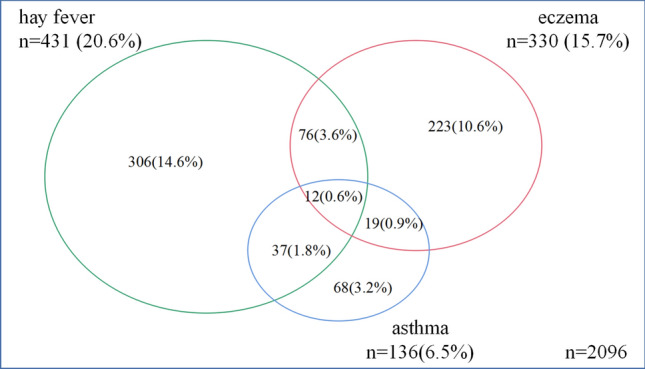
Distribution of eczema, hay fever, and asthma among the study cohort

A total of 2096 adults in six regions of the northern grassland of Mongolia were enrolled and completed the study. There were 850 (40.5%) male and 1246 (59.5%) female subjects in the study cohort. Overall, 330 subjects were found to have eczema, with a prevalence of 15.7% (95% CI 14.3–17.4, Table 1). The prevalence of hay fever was 20.6% (95% CI 18.9–22.4) among individuals with eczema, and 6.5% (95% CI 5.5–7.6) among those with asthma. The prevalence of hay fever and/or asthma was 24.7% (95% CI 22.9–26.1, Fig. 3). In particular, 5.1% (95% CI 4.2–6.1) of the adult population was diagnosed with EAH.

**Table 1:** Demographic characteristics of the eczema and EAH study population

Variables	Eczema			EAH			Total
	No (n = 1766)	Yes (n = 330)	*P*	No (n = 1989)	Yes (n = 107)	*P*	
Age (years), mean ± SD	47.94 ± 14.85	43.32 ± 14.02	0.001	48.22 ± 13.89	44.99 ± 13.65	< 0.001	47.71 ± 13.90
Gender, n (%)			0.698			0.312	
Male	713 (83.9)	137 (16.1)		812 (95.5)	38 (4.5)		850 (40.6)
Female	1053 (84.5)	193 (15.5)		1177 (94.5)	69 (5.5)		1246 (59.4)
Ethnicity, n (%)			0.811			0.645	
Han	1050 (84.3)	195 (15.7)		1182 (94.9)	63 (5.1)		1245 (59.4)
Mongolian	649 (84.4)	120 (15.6)		731 (95.1)	38 (4.9)		769 (36.7)
Other	67 (81.7)	15 (18.3)		76 (92.7)	6 (7.3)		82 (3.9)
Residence, n (%)			< 0.001			< 0.001	
Rural	857 (80.6)	206 (19.4)		988 (92.9)	75 (7.1)		1063 (50.8)
Urban	907 (88.0)	124 (12.0)		999 (96.9)	32 (3.1)		1031 (49.2)
Education level, n (%)^a^			< 0.001			< 0.001	
Low	457 (90.5)	48 (9.5)		496 (98.2)	9 (1.8)		505 (24.1)
Medium	796 (86.3)	126 (13.7)		893 (96.9)	29 (3.1)		922 (44.1)
High	511 (76.7)	155 (23.3)		598 (89.8)	68 (10.2)		666 (31.8)
Marital status, n (%)^b^			0.411			0.307	
Unmarried	120 (80.5)	29 (19.5)		138 (92.6)	11 (7.4)		149 (7.2)
Married	1601 (84.6)	291 (15.4)		1801 (95.2)	91 (4.8)		1892 (90.9)
Divorced/widowed	35 (85.4)	6 (14.6)		38 (92.7)	3 (7.3)		41 (2.0)
Annual family income (CNY, mean ± SD)^c^	5.1 ± 4.0	5.7 ± 5.3	0.007	5.1 ± 4.1	6.5 ± 4.6	0.001	5.2 ± 4.2
BMI^d^, n (%)			0.608			0.409	
Lean or healthy	873 (83.7)	170 (16.3)		983 (94.2)	60 (5.8)		1043 (49.8)
Overweight	696 (86.3)	129 (15.6)		788 (95.5)	37 (4.5)		825 (39.4)
Obese	196 (86.3)	31 (13.7)		217 (95.6)	10 (4.4)		227 (10.8)
Family history, n (%)			< 0.001			< 0.001	
Positive	579 (32.8)	147 (44.5)		663 (33.3)	63 (58.9)		726 (35.7)
Negative	1187 (67.2)	183 (55.4)		1326 (66.7)	44 (41.1)		1310 (64.3)

^a^Education was categorized as low (received only primary education or no education), medium (finished secondary school or high school) and high (graduated from college or university). Education status was missing in 3 cases in this study

^b^Marital status was missing in 14 cases

^c^Family income was calculated as 10,000 RMB/year/ family

^d^BMI was categorized according to the World Health Organization criteria, with BMI < 25 kg/m^2^ defined as lean or healthy, BMI between 25 and 29 kg/m^2^ defined as overweight, and BMI ≥ 30 kg/m^2^ defined as obese. BMI status was missing in 1 case

### Associations with eczema and EAH

**Fig. 4:**
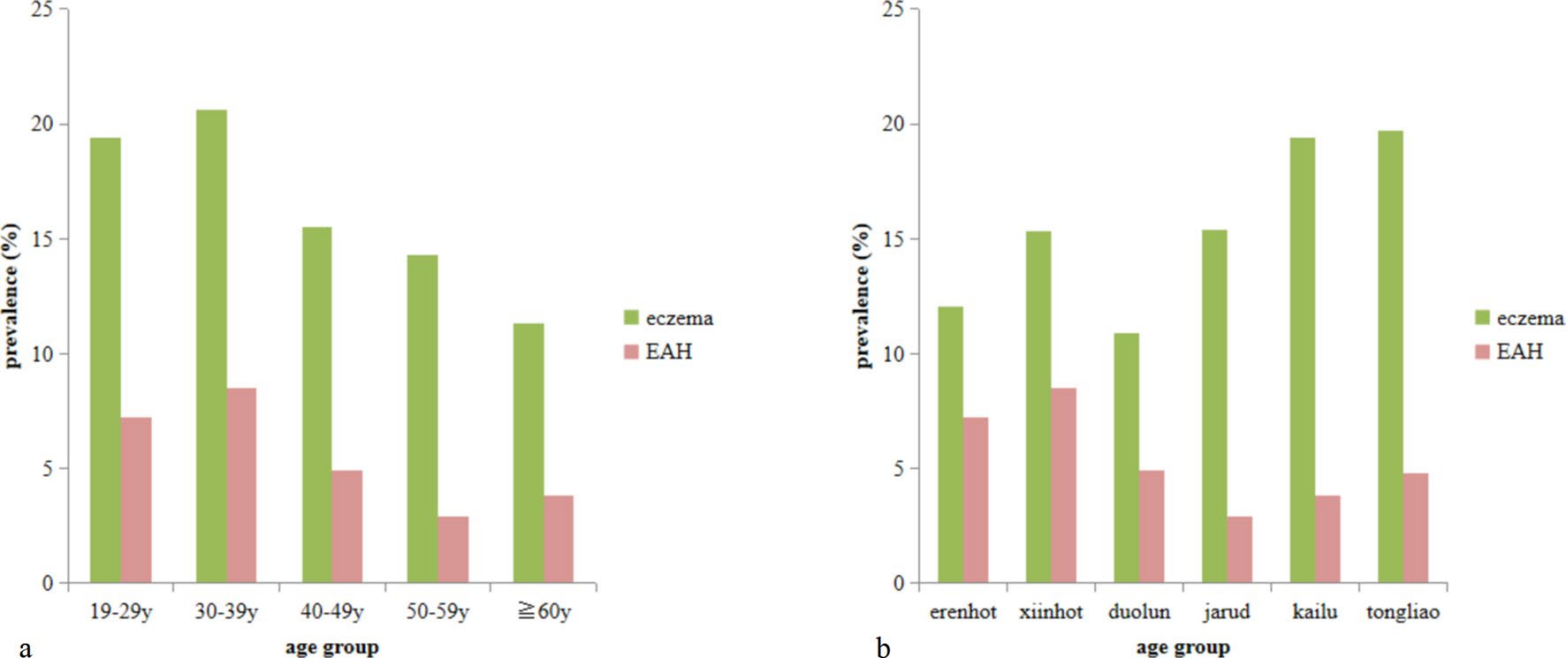
Variation in eczema and EAH prevalence across different age groups and geographic regions

The prevalence of adult eczema (19.4% vs 12.0%, *P* < 0.001) and EAH (7.1% vs 3.1%, *P* < 0.01) was higher in urbanized areas compared with rural areas (Table 1). No gender or ethnic differences were found in the prevalence of eczema and EAH. The prevalence of eczema and EAH showed significant difference among age groups and was highest in the 30–39 year group (20.6% and 8.5%, respectively) (*P* = 0.003, *P* = 0.001, respectively). Prevalence of positive family history of atopy was higher in EAH group than in eczema group (58.9% vs 44.5%, *P* < 0.001). There was a significant difference in the prevalence of eczema among the six study areas (*P* = 0.007), but EAH did not vary significantly (*P* = 0.32) (Fig. 4).

Socioeconomic factors that were associated with higher prevalence of adult eczema and EAH are shown in Table 1. In particular, a higher level of education (*P* < 0.001, both) and higher family income (*P* = 0.007 and *P* < 0.001, respectively) were associated with increased prevalence of eczema and EAH. Environmental factors are evaluated in Table 2, which shows that the prevalence of EAH was significantly different depending on heating mode (*P* = 0.029); however, this variation was not seen in eczema (*P* = 0.073). Antibiotic overuse significantly increased the prevalence of EAH (9.0% vs 4.1%, *P* < 0.001), but did not increase the prevalence of eczema (*P* = 0.235).

**Table 2:** The environmental determinants of the eczema and EAH study population

Variables, n (%)	Eczema			EAH		
	No (n = 1766)	Yes (n = 330)	*P*	No (n = 1989)	Yes (n = 107)	*P*
Pet keeping			0.188			0.330
No	1379 (83.7)	268 (16.3)		1559 (94.7)	88 (5.3)	
Yes	386 (86.4)	61 (13.6)		429 (96.0)	18 (4.0)	
Smoking habit			0.259			0.354
Never	1301 (84.5)	238 (15.5)		1455 (94.5)	84 (5.5)	
Current	325 (85.1)	57 (14.9)		368 (96.3)	14 (3.7)	
Stopped	117 (79.6)	30 (20.4)		140 (95.2)	7 (4.8)	
Heating mode			0.073			0.029
Wood heating	261 (88.8)	33 (11.2)		288 (98.0)	6 (2.0)	
Coal heating	215 (84.3)	40 (15.7)		244 (95.7)	11 (4.3)	
Central heating	1206 (83.5)	239 (16.5)		1363 (94.3)	82 (5.7)	
Antibiotic overuse (≥ 3 times/years)			0.235			< 0.001
No	1364 (85.1)	239 (14.9)		1538 (95.9)	65 (4.1)	
Yes	368 (82.7)	77 (17.3)		405 (91.0)	40 (9.0)	
Daily outdoor activity			0.133			0.857
≤ 1 h	276 (81.9)	61 (18.1)		319 (94.7)	18 (5.3)	
2–3 h	836 (83.6)	164 (16.4)		947 (94.7)	53 (5.3)	
≥ 4 h	653 (86.3)	104 (13.7)		721 (95.2)	36 (4.8)	

*EAH* Eczema with asthma and/or hay fever

The prevalence of eczema was higher in subjects with a higher education level (OR 2.49, 95% CI 1.55–4.04, *P* < 0.001 in medium compared with low level) while medium and high education levels increased the risk of EAH (OR 3.31, 95% CI 1.83–5.98, *P* < 0.001 and OR 5.38, 95% CI 2.04–14.18, *P* < 0.001) as shown by a multivariate logistic regression model. Family history of atopy increased eczema risk 1.39-fold, and increased EAH risk 2.10-fold (*P* = 0.017,* P* = 0.002 respectively). Antibiotic overuse increased the risk of EAH, but not eczema (OR 1.71, *P* = 0.022 and OR 0.95, *P* = 0.756, respectively) (Table 3).

**Table 3:** Common risk factors among subjects with adult eczema and EAH by multivariate logistic regression

Variable	Eczema			EAH		
	OR	95% CI	*P* value	OR	95% CI	*P* value
Age (years), quartile						
1 (18–37)	1	1	–	1	1	–
2 (38–47)	1.57	1.00–2.46	0.052	0.80	0.37–1.74	0.579
3 (48–60)	1.58	1.01–2.48	0.044	0.91	0.42–1.98	0.810
4 (≧ 60)	1.36	0.88–2.10	0.169	1.46	0.64–3.33	0.357
Residence						
Rural	1	1	–	1	1	–
Urban	1.39	1.01–1.92	0.045	1.27	0.71–2.27	0.423
Education level						
Low	1	1	–	1	1	–
Medium	2.49	1.54–4.04	< 0.001	3.31	1.83–5.98	< 0.001
High	1.45	0.96–2.19	0.078	5.38	2.04–14.18	< 0.001
AFI^a^, quartile						
1 (0–2)	1	1	–	1	1	–
2 (3–4)	1.20	0.80–1.81	0.386	0.90	0.46–1.76	0.747
3 (5–7)	1.14	0.77–1.67	0.520	1.78	0.89–3.54	0.104
4 (≥ 8)	1.15	0.80–1.64	0.450	1.46	0.82–2.59	0.199
Family history						
No	1	1	–	1	1	–
Yes	1.39	1.06–1.82	0.017	2.10	1.33–3.34	0.002
Antibiotics use (> 3/year)						
No	1	1	–	1	1	–
Yes	0.95	0.70–1.29	0.756	1.71	1.08–2.71	0.022
Heating mode						
Wood heating	1	1	–	1	1	–
Coal heating	1.36	0.86–2.15	0.194	0.73	0.26–2.06	0.547
Central heating	1.43	0.94–2.18	0.098	1.34	0.50–3.57	0.561

^a^AFI: annual family income, 10,000 RMB/year

### Allergen sensitization in adult eczema and EAH

**Fig. 5:**
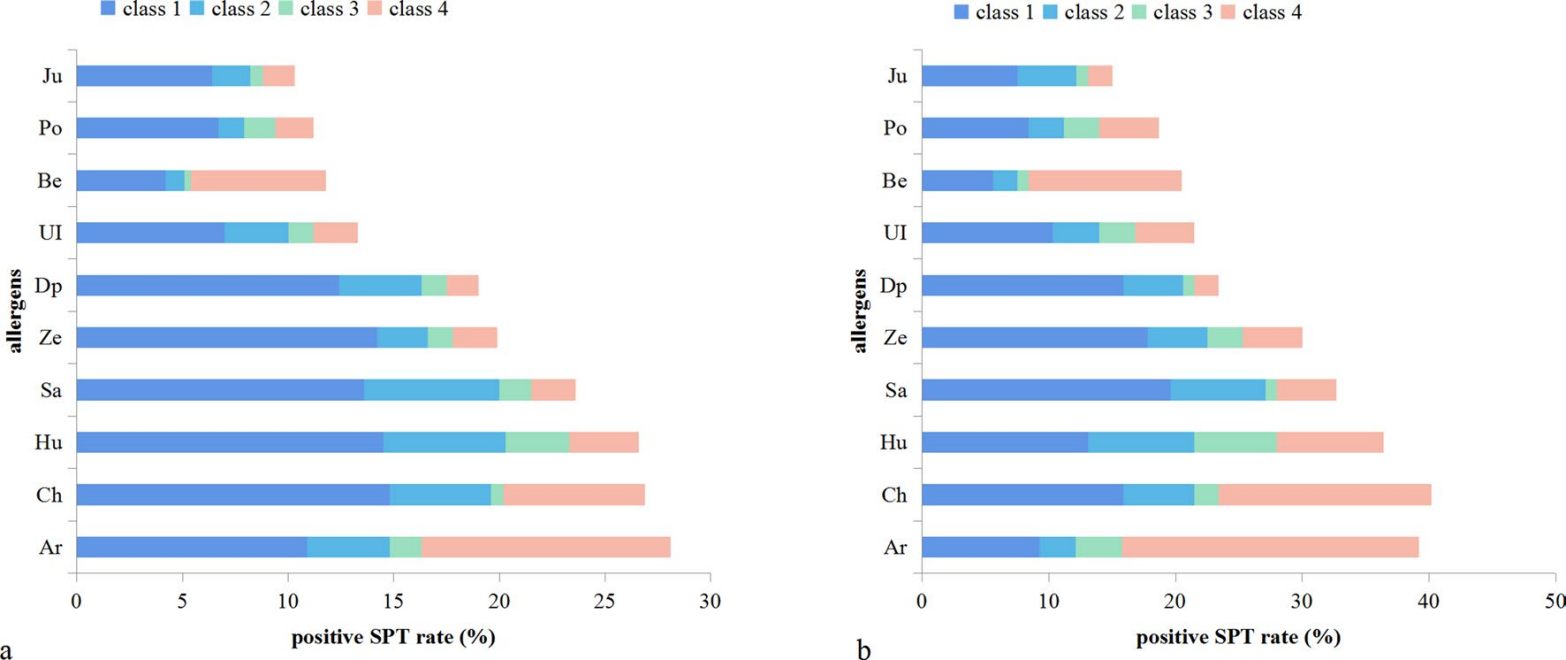
The sensitizati›on rate from SPT in eczema and EAH groups. **a** Demonstrates the sensitization rate in the eczema group, while **b** demonstrates the sensitization rate of the EAH group

The aeroallergens sensitization rate was 48.2% in the eczema group and 41.0% in the non-eczema subjects (*P* = 0.018). Regarding EAH, the aeroallergen sensitization rate was 53.8%, which was higher than the eczema population (*P* < 0.001). The sensitization rates to 10 aeroallergens are shown in Fig. 5, with the most common being weed pollen sensitization. The positive rate of Dp was 23.4% in EAH and 19.0% in eczema (*P* < 0.001). The sensitization of Dp was positively correlated with increasing eczema (OR 1.52, 95% CI 1.12–2.07, *P* = 0.007) and EAH (OR 1.90, 95% CI 1.19–3.03, *P* = 0.007) (Table 4). Allergen sensitization could increase the risk of eczema and EAH which was more obvious with Dp than pollen (OR 1.52 vs 1.29 in eczema and OR 1.90 vs 1.82 in EAH, respectively).

**Table 4:** The associations of allergic diseases and allergic sensitization with eczema/EAH

Characteristics	n	Eczema			EAH#		
		*P*	OR	95% CI	*P*	OR	95% CI
Hay fever	431	0.003	1.51	1.15–1.98	–	–	–
Allergic rhinitis	746	< 0.001	1.57	1.23–1.99	< 0.001	22.53	11.31–44.89
Allergic conjunctivitis	775	< 0.001	1.57	1.24–1.99	< 0.001	3.45	2.29–5.19
Asthma	136	0.02	1.64	1.49–2.08	–	–	–
Food allergy	276	< 0.001	2.86	2.14–3.82	< 0.001	2.76	1.77–4.30
Drug allergy	446	0.001	1.57	1.20–2.05	0.047	1.55	1.01–2.38
Disease kinds							
One disease	608	< 0.001	1.83	1.33–2.52	0.001	2.17	1.35–3.49
Two diseases	453	< 0.001	1.94	1.38–2.73	< 0.001	5.22	3.03–8.98
≥ 3 diseases	260	< 0.001	3.66	2.56–5.24	< 0.001	27.32	11.38–65.58
SPT positive							
Pollen	843	0.035	1.29	1.02–1.64	0.003	1.82	1.23–2.68
HDM	300	0.007	1.52	1.12–2.07	0.007	1.90	1.19–3.03
Pollen + HDM	883	0.015	1.34	1.06–1.69	0.002	1.88	1.27–2.79

^a^Hay fever and asthma were not included when evaluating the associations of allergic diseases and disease kinds with EAH

### The association of allergic diseases with eczema and EAH

Allergic disease comorbidity could increase the risk of eczema and EAH (Table 4). In particular, food allergy was associated with eczema (OR 2.86, 95% CI 2.14–3.82, *P* < 0.001) and EAH (OR 2.76, 95% CI 1.77–4.30, *P* < 0.001). Subjects with two or more combined allergic diseases were at increased risk of having eczema and EAH (*P* < 0.001).

## Discussion

Eczema represents a group of conditions with no standard clinical definition. The diagnosis of adult AD can be challenging since this terminology has been defined recently and a lack of familiarity and confusion in the definition of the condition persists [6–8]. As in other studies, two definitions were applied in the present study in order to capture all the features of the eczema/AD disease spectrum [17, 22]. The term “eczema” describes a host of ill-defined skin lesions, and encompasses the acute, subacute, and chronic lesions present in AD [8]. However, use of such an imprecisely defined term as eczema could result in an overestimation of the actual prevalence of AD. In contrast, the term “EAH,” while more specific at identifying AD, may result in an underestimation of the prevalence of true AD. In the present study, EAH was assumed to be the proxy indicator of AD.

Adult AD has distinct clinical characteristics as compared to child-onset AD [6]. Herein, we found a higher prevalence of adult eczema (15.7%) and EAH (5.1%) in the northern grassland region of China compared to reports from the US and Europe [17, 22, 29]. The US National Health Interview Survey reported the prevalence of adult eczema as 10.2% and EAH as 3.2% [22]. In Italy, the prevalence of eczema and EAH were 8.1% and 3.4%, respectively, among 10,464 adults [17]. Besides those studies, other studies used different diagnostic criteria, hence the comparability was unreliable. An international, cross‐sectional, web‐based survey of adults with AD was performed using modified UK Working Party/ISAAC criteria, and self‐report history revealed an overall adult prevalence of AD of 4.9% [21]. Although the prevalence was approximately the same as our study, the different survey measurement approach (online vs field-interview) made it hard to compare. A study in Japan noted the prevalence adult AD to be 3% as defined by the UK Working Party [30]. In Sweden, the prevalence of self-reported eczema as defined by GA2 LEN questionnaire was 11.5% in 2012 [18]. These surveys provide different accounts of epidemiological features of adult AD and may improve our understanding of adult AD in different regions. In China, few surveys have been carried out, and those that have been conducted focused primarily on children. A survey of 11,473 school children from two areas in Guangdong noted the prevalence of eczema and EAH as 25.9% and 34.1%, respectively [31]. In Shanghai, the prevalence of childhood AD was 8.4% in 2016 [24]. A nationwide study of adolescents in China found AD in 2.5% of subjects [25]. The higher prevalence of adult eczema and AD in our study suggest that AD is increasing in select geographic areas perhaps secondary to elevated pollen in these areas. This study suggested that adult AD is more common than previously thought. Of note, variation in methods and societal norms may also contribute to study variation. AD is regarded mainly as a childhood disease and can be outgrown with age. While this may be historically the case, recent studies indicate that AD is not restricted to children. In Italy, over 50% of individuals with AD reported adult onset [17]. This could account, in part, for the high prevalence of AD in our study.

The variation of adult AD prevalence suggests a role for socioeconomic and environmental factors. No gender difference was found in the present study in contrast to other reports that noted women displayed the study-defined symptomology and clinical presentations [6, 29]. Peak prevalence was most frequently observed in the 29–39 year age‐groups and decreasing prevalence was observed in the ≥ 40 year age‐groups in our study, which is consistent with an international survey of adult AD [21]. Urbanization was associated with an increased prevalence of AD, which is consistent with previous studies [16, 18]. Taken together, these data suggest that rapid urbanization and industrialization may be involved in the progression of AD. This phenomenon is explained by the hygiene hypothesis, which postulates that environmental antigens in early life is paramount in the development of the immune system [32]. Urbanization and industrialization likely decrease the early exposure to antigens, thus resulting in immune dysfunction and atopic diseases. Another explanation is the increasing exposure to airborne pollution. Pollutants such as NO_2_, heavy traffic and exposure to diesel exhaust could damage the skin barrier, increase trans-epidermal water loss, and increase the production of IgE [33–36]. Environmental and socioeconomic determinants were analyzed in this study. Several risk factors were found to be associated with eczema/AD, including higher education levels and household incomes. These results suggested an increase of AD with increasing socioeconomic status both in adults and children [33–36]. Higher socioeconomic status may lead to a reduction in allergen exposure in early life. Place of birth and ethnicity could also contribute to the increasing frequency of AD [15, 22, 37–39]. Antibiotic overuse was found to be associated with increasing prevalence of allergic rhinitis and asthma in our previous study [40]. In this study, antibiotic overuse was associated with EAH but not eczema, which could be explained by the different subsets of the two diseases. EAH is a typical atopic disease with allergen sensitization contributed to its pathogenesis while eczema was more complicated with less atopic factors involved. Exposure to antibiotics could alter immune balance and favor over-activity that manifests as allergic reaction. In this study, subjects who used wood or coal heating had a higher prevalence of AD than those who used central heating. This effect may be explained by dry air impairing skin barrier function due to central heating and less outdoor activity [32]. A lower prevalence of AD was significantly associated with outdoor humidity, higher temperature, UV light exposure, and less indoor heating [13, 41]. In Inner Mongolia, it is presumed that similar climate factors may influence the prevalence of adult eczema/AD, such as low temperature and arid weather conditions. In brief, younger age, atopy family history, high education level, urbanization, and antibiotic overuse were found to be the risk determinants. Although the prior four are well-known, the impact of antibiotics on AD is relatively new and should be of concern.

Another key topic addressed in this study was the comorbidities of adult AD. A higher prevalence of other allergic diseases, such as asthma and hay fever, was found in the eczema/AD group compared with the non-eczema group in our study. Thus, AD may play a role in the persistence of asthma or hay fever [1, 3, 9]. Recently, a genome-wide association study [42] of a broad allergic disease phenotype found that 130/136 gene variants were similar in individuals with asthma, hay fever, and AD, thus strongly supporting a genetic aspect to these conditions. Inherited and acquired factors lead to abnormal epidermal structure and function, which could be the beginning of an “allergy march”. Studies showed that epigenetic pathways could mediate the gene × environment interactions and impact the “allergy march” [43]. Epigenetic markers, such as DNA methylation, have been shown to demonstrate a potential for the development of diagnostic tools for allergies [43, 44]. Besides atopic comorbidities, several chronic conditions are associated with adult AD including diabetes, psychiatric diseases, cardiovascular diseases, bone fracture, and hypertension [5, 45–49]. Adult AD should be considered as a systemic disease rather than a skin disease.

One strength of this study was the evaluation of allergic sensitization for adult AD. It is intriguing that the prevalence of AD is highly affected by aeroallergen sensitization. High pollen concentration, as we reported [26], may contribute to the development of AD in grassland habitats. Typically, pollen could induce nasal or asthmatic symptoms rather than dermatological symptom. But under extremely high pollen exposure, prevalence of AD could be high. Interestingly, we found that house dust mite (HDM) allergy had a lower prevalence in subjects from grasslands and in allergic rhinitis subjects. Conversely, HDM allergy was associated with a higher positive SPT rate than tree pollen and grass pollen in AD subjects. Thus, we speculated that two reasons may contribute to this phenomenon. First, the impact of pollen on AD may be determined by pollen exposure concentrations. In northern grassland region, weed pollen has a higher concentration than tree/grass pollen and could induce more eczema/AD than other type of pollen. Second, HDM allergy was more important in AD rather than allergic rhinitis or other allergic conditions. Subjects sensitized to HDM has a higher risk of developing eczema/AD despite low concentration exposure. However, whether the prevalence and comorbidities of eczema and EAH could change under lower pollen exposure or higher HDM exposure will need further investigation.

We performed a population-based investigation and analyzed the allergen sensitization of eczema/AD. Efforts to minimize selection bias were made. However, some limitations should be mentioned. First, eczema was defined as self-reported cases of doctor-diagnosed “eczema” or “atopic dermatitis”. This might overestimate the prevalence of adult eczema due to possible confusion with irritant or contact dermatitis. Second, the prevalence of adult AD may include both childhood persistent AD or adult-onset AD [28]. In this study, we did not list the onset age of AD nor classify the subtype of adult AD. Third, the duration of AD, the severity of AD, and the lesion site of AD were not mentioned in this study. Thus, it is impossible to provide detailed phenotypic information about adult AD. Finally, food allergen sensitization which was closely related with AD was not included in this study. Thus, assessment of the role of allergy in eczema/AD was incomplete. Further studies concerning the impact of food allergen sensitization, lower pollen exposure or higher HDM exposure on AD should be conducted in the future to understand full aspects of AD.

## Conclusions

The prevalence of adult eczema and AD in the northern grassland area of China was 15.7% and 5.1%, respectively. Adult eczema/AD was positively associated with increased asthma and hay fever. Significant predictors of adult eczema/AD included age, place of residence, higher education level, higher family income, family history, heating mode, overuse of antibiotics, allergen sensitization, and comorbid atopic diseases.

## Data Availability

The datasets used and/or analyzed during the current study are available from the corresponding author on reasonable request.
